# Red and Red Processed Meat Consumption Behaviors in Scottish Adults

**DOI:** 10.1016/j.cdnut.2024.103777

**Published:** 2024-05-16

**Authors:** Cristina Stewart, Ricki Runions, Geraldine McNeill, Fiona Comrie, Alana McDonald, Lindsay M Jaacks

**Affiliations:** 1 Global Academy of Agriculture and Food Systems, University of Edinburgh, Midlothian, United Kingdom; 2 MRC/CSO Social and Public Health Sciences Unit, School of Health and Wellbeing, University of Glasgow, United Kingdom; 3 Food Standards Scotland, Aberdeen, United Kingdom

**Keywords:** meat consumption, meat consumption behaviors, beef, pork, processed meat, Scottish Health Survey, Scotland, UK, sustainable diets

## Abstract

In 2021, 32% of adult meat consumers in Scotland exceeded the 70 g/d recommended limit of red and red processed meat (RPM) intake. We analyzed RPM consumption behaviors among adults in the Scottish Health Survey (2021) to better understand this consumer group. Consumers were categorized into low, medium, and high consumers, and overall, mean intake was 66 g/d. Males and individuals living in the most deprived areas were most likely to be high consumers (45% compared with 30% for males compared with females, respectively, and 44% compared with 32% for those in the most compared with least deprived areas, respectively). Dinners accounted for the majority of intake among high (55%) and medium (52%) consumers, whereas low consumers distributed intake between lunch (40%) and dinner (48%). Across all groups, consumption was highest on Sundays, and majority of RPM was purchased at supermarkets. Beef dishes and sandwiches were primary contributors among high and medium consumers. These insights can inform the design of effective strategies and policies aligned with meat reduction targets. For instance, strategies focusing on modifying traditional meat-centric dishes and sandwiches could be impactful.

## Introduction

High consumption of processed meat, and to a lesser extent unprocessed red meat, is associated with increased risks of several noncommunicable diseases including type 2 diabetes, cardiovascular disease, and colorectal cancer [[1], [2], [3]]. In 2015, the WHO classified processed meat as a carcinogen, and red meat as a probable carcinogen [2]. Further, the links between red and red processed meat (RPM) intake and colorectal cancer led to the UK Scientific Advisory Committee on Nutrition recommending that adults with high intakes (>90 g/d) consider reducing their intake to a maximum of 70 g/d [4]. Based on this advice, Scottish Government set this target as a Scottish Dietary Goal in 2016 [5].

Meat production, particularly ruminant meat (i.e., beef and lamb), is also a leading contributor to environmental degradation [6]. This evidence led to the UK Committee on Climate Change (CCC) recommending in 2020 that consumption of beef, lamb, and dairy in the UK decreases by ≥20% by 2030 [7]. In their 2022 report, the CCC recommended the Scottish Government “take low-cost, low-regret actions to encourage a 20% shift away from all meat by 2030, rising to 35% by 2050” [8], which the Scottish Government have partially accepted [9]. However, these targets are modest, and more ambitious targets exist. For instance, the EAT–Lancet commission recommended >50% reduction in global red meat consumption and suggest intake should be limited to 0–28 g/d [10]. Another study based on micronutrient adequacy of intake recommended an additional 6 g/d of pork, which would increase the upper end of the range to 34 g/d [11].

In 2018–2019, more than a third (34%) of adult meat consumers in the United Kingdom exceeded the 70 g/d RPM recommendation, with consumers eating a mean intake of 35 g/d unprocessed red meat and 36 g/d processed meat [12]. Similar figures were observed in Scotland in 2021, with 32% of adult meat consumers exceeding the recommendation [13]. Supporting individuals to reduce their meat consumption in line with these targets is important for improving both population and environmental health.

This study investigated consumption behaviors of RPM among adults in Scotland. By categorizing consumers into low, medium, and high consumer groups, this study estimated current RPM intakes, examined demographics, and explored the following 3 aspects related to its consumption across these consumer groups: *1*) the timing of consumption (day of the week and meal occasion); *2*) the locations where RPM is purchased; and *3*) how RPM is consumed, focusing on food group contributors to intake.

## Methods

Data from adults (≥16 y) in the 2021 nationally representative cross-sectional survey, the Scottish Health Survey (SHeS), were analyzed. SHeS 2021 was granted ethical approval by the Health and Care Research Ethics Committee for Wales (REF: 17/WA/0371). Detailed SHeS methodology is provided elsewhere [14]. Dietary data were from 1–2, online, self-administered 24-h dietary recalls (https://intake24.org).

Estimates of meat intake were based on disaggregated values, where all non-meat components of composite dishes were excluded [15]. We used the International Agency for Research on Cancer’s definition of processed meat—that is, meat that has been transformed through salting, curing, fermentation, smoking, or other processes to enhance flavor or improve preservation. With this, RPM included the following meat subtypes disaggregated in SHeS: beef, lamb, pork, other red meat, processed red meat, burgers, sausages, and offal. Processed white meat (contributing 2% of all processed meat consumed) was not included (Supplemental Table 1). We grouped consumers (i.e., those consuming >0 g of RPM across their recalls) into groups based on their mean daily intake.

Respondents assigned a meal name to each meal they reported in their recalls, allowing us to analyze consumption by breakfast, lunch, dinner, and snacks (Supplemental Methods). Respondents also reported where they purchased each meal (or the ingredients), and with this, we estimated consumption by purchase location (e.g., supermarkets, cafés, restaurants, and takeaways) (Supplemental Methods). We estimated mean intakes (in both grams and percent contribution to total intake) per meal occasion, and purchase location, by summing each respondents’ intakes across all recalls and dividing by the number of completed recalls. We also estimated intakes by day of the week, with majority of estimates based on single recalls (i.e., those completing only 1 recall or for those who completed 2 recalls on different days of the week). However, 3% of consumers (*n* = 82) completed 2 recalls on the same day of the week (across different weeks), and an average was calculated across their 2 recalls. As such, only absolute intakes (in grams) for each day of the week were calculated, not the percent contribution to intake.

We established food group contributors to RPM intake overall and among consumer groups across 3 levels: high-level food category (e.g., meat and meat products), main food group (e.g., beef dishes), and sub food group (e.g., manufactured beef products). All food groups were analyzed and reported in line with the UK National Diet and Nutrition Survey definitions [16].

All results were survey weighted, and all dietary recalls were included in the analyses. We descriptively explored how demographics [based on gender, age group, and the Scottish Index of Multiple Deprivation (SIMD)] differed across consumer groups. SIMD is an official measure of area-based deprivation, based on a range of census-based indicators across income, employment, housing, health, education, skills and training, and access to services. We used a multivariable logistic regression model to determine whether being a consumer differed by demographic subgroup and a multivariable ordinal logistic regression to test for differences in consumer group by subgroups. Both models adjusted for mean daily energy intake. Analyses were carried out using Stata IC, version 17, and figures were created in R, version 4.1.2. Statistical significance was set at *P* < 0.05.

## Results

Of the 4557 respondents aged ≥16 y, 3447 completed ≥1 recall (unweighted 76%), of which the majority (*n* = 2494; unweighted 72%) consumed some quantity of RPM, comprising our final sample. Consumers were, on average, 53.7 y, 51% were male, and 32% were aged 35–54 y (Supplemental Data).

Consumers were categorized into low (*n* = 824; >0 to ≤34.5 g/d), medium (*n* = 784; >34.5 to 70 g/d), and high (*n* = 886; >70 g/d) consumers. We created 3 equal sized groups, and the cutoff for high consumers was 72 g/d, which we adjusted to ensure alignment with the recommended limit of 70 g/d, affecting 43 respondents.

Of consumers who completed 2 recalls (*n* = 2269), 50% consumed RPM on both days (16% of low, 52% of medium, and 76% of high consumers). Mean intake of RPM was 66 g/d (95% CI: 64, 69) among all consumers; 19 g/d (95% CI: 18, 20) among low consumers; 50 g/d (95% CI: 49, 51) among medium consumers; and 117 g/d (95% CI: 112, 121), among high consumers. Overall, the majority of RPM (56%) was processed (57% among low, 58% among medium, and 54% among high consumers), and most processed meat was pork (87%) (Supplemental Data).

There was no difference in odds of being a consumer by age group or SIMD although males were more likely to be a consumer (77%) than females [70%; odds ratio (OR): 1.26; 95% CI: 1.02, 1.55; *P* = 0.029], when adjusting for mean daily energy intake. Further, males were more likely to be a high consumer (45%) than females (30%; OR: 1.66; 95% CI: 1.39, 1.98; *P* < 0.001). Those in SIMD 5 (least deprived) were less likely to be a high consumer (32%) than those in SIMD 1 (most deprived; 44%; OR: 0.62; 95% CI: 0.46, 0.83; *P* = 0.001).

**FIGURE 1:**
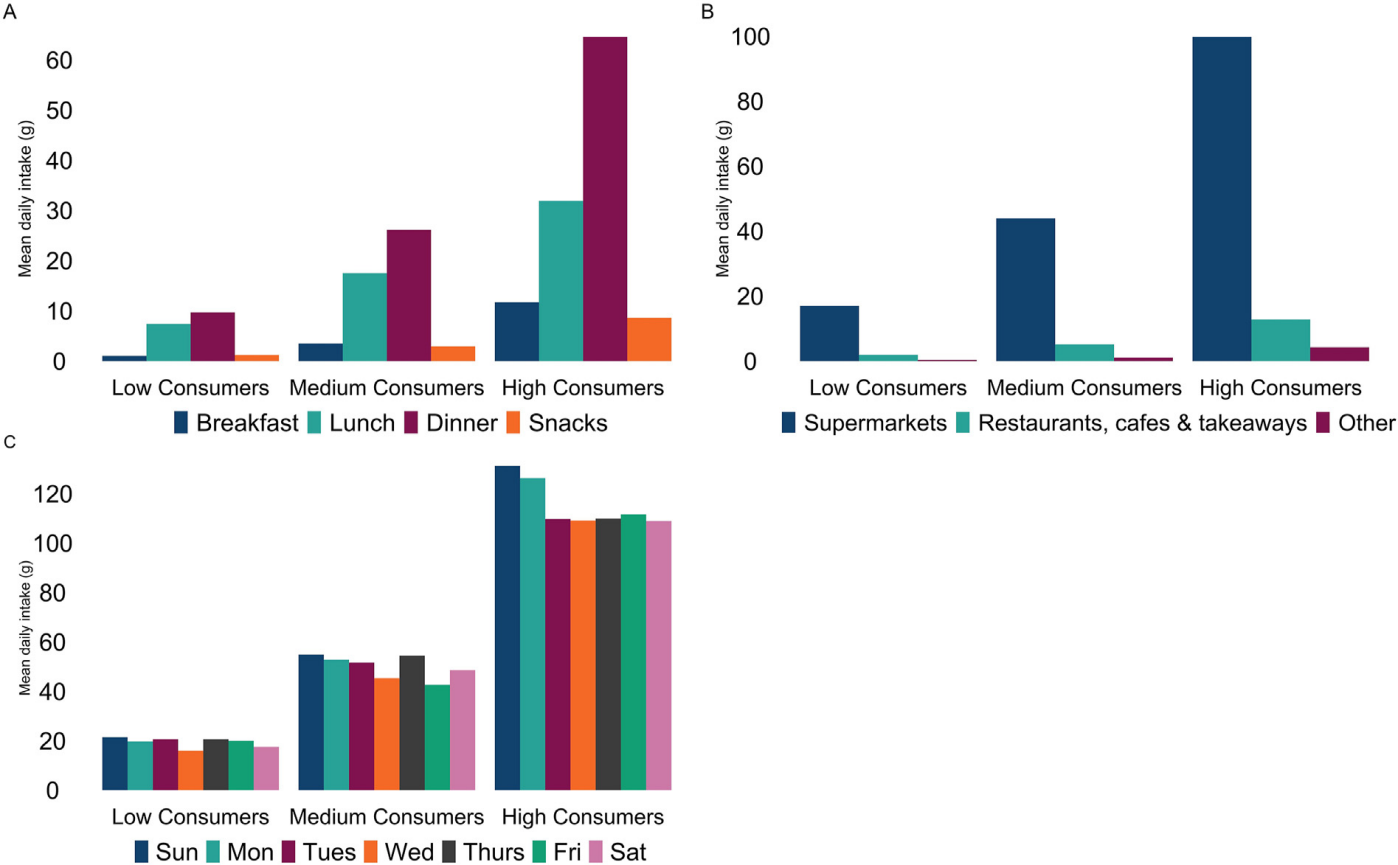
Mean daily intakes (g) of red and red processed meat by (A) meal occasion; (B) purchase location; and (C) day of the week, by consumer group. Low consumers (*n* = 824; >0 to ≤34.5 g/d); medium consumers (*n* = 784; >34.5 to 70 g/d); and high consumers (*n* = 886; >70 g/d). Percent contributions are available in Supplemental Data.

**FIGURE 2:**
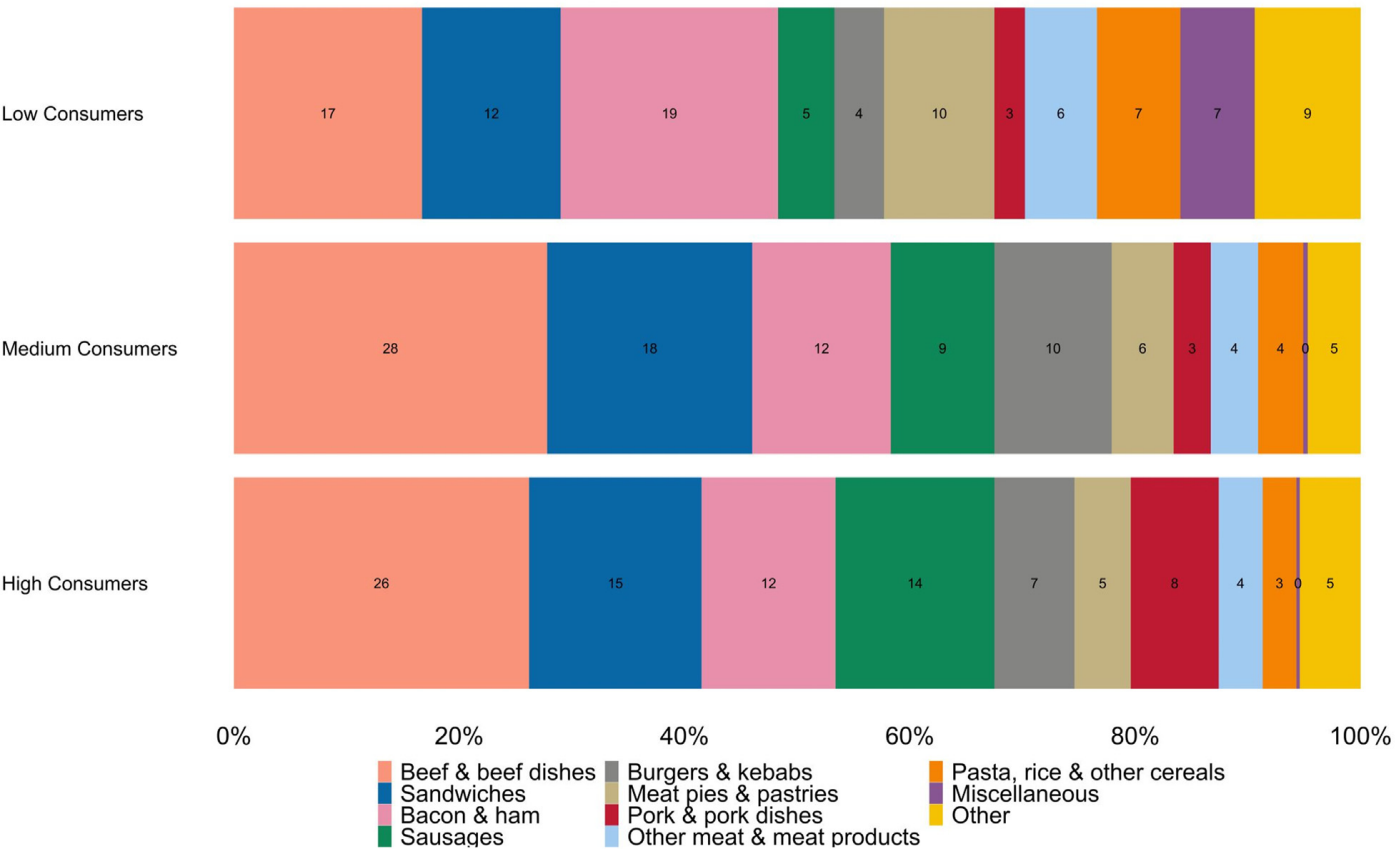
Percent contribution of main food groups to red and red processed meat intake by consumer group. Low consumers (*n* = 824; >0 to ≤34.5 g/d); medium consumers (*n* = 784; >34.5 to 70 g/d); and high consumers (*n* = 886; >70 g/d). Food groups contributing <5% to all groups are categorized into other. Percent contribution of miscellaneous items to medium and high consumers was 0.4% and 0.3%, respectively. Absolute intakes (in grams) and percent contributions from all food groups contributing ≥0.5% to total red and red processed meat intake are available in Supplemental Data.

Overall, dinners accounted for the highest proportion of RPM intake (52%), as well as among high (55%) and medium (52%) consumers. Low consumers distributed their intake more across lunch (40%) and dinner (48%) (Figure 1). The majority of RPM consumed was purchased from supermarkets (85%–88% across consumer groups), with 10%–12% purchased from cafes, restaurants, and takeaways (Figure 1). Overall, and across consumer groups, intake was highest on Sundays (Figure 1). Beef dishes and sandwiches [notably spaghetti Bolognese and ham sandwiches (Supplemental Data)] were the largest contributors to intake among high (26% and 15%, respectively) and medium (28% and 18%, respectively) consumers. Bacon and ham, followed by beef dishes, were the largest contributors among low consumers (19% and 17%, respectively) (Figure 2). Detailed intake results are provided in Supplemental Data.

## Discussion

To our knowledge, this is the first study examining the situational contexts of RPM consumption within a nationally representative survey. Half of adults in Scotland consumed RPM on both recall days, with high consumers averaging 117 g/d. Males and individuals living in the most deprived areas were more likely to be high consumers than females and those living in the least deprived areas. Dinners accounted for the majority of RPM intake, with intake highest on Sundays, and most RPM being purchased at supermarkets. Beef dishes and sandwiches were the largest contributors to RPM intake among high consumers, whereas low consumers were primarily eating RPM through bacon and ham and beef dishes.

Our findings align with previous research showing higher RPM consumption among males and individuals living in the most deprived areas, in both Scotland and the wider United Kingdom [[17], [18], [19]]. Our results also suggest that low consumers have more diverse meat consumption patterns than high consumers, resulting in a wider variety of food groups contributing to meat intake. High consumers had greater contributions from beef dishes, pork dishes, burgers, and kebabs than low consumers. We can speculate that they may have a more traditional preference toward meat-centric meals, perhaps rooted in established meat-eating habits. Conversely, low consumers had greatest contributions from bacon and ham, which may be more versatile and integrated into a broader range of meals. Interestingly, a Dutch-based study found one of the most common reasons for low-meat eaters not eating meat frequently was because they preferred to vary their meals [20]. In this study, low consumers had higher contributions from meat pies and pastries, other manufactured meat products, and pizza, than high consumers. This contradicts other research suggesting low consumers (or indeed meat-reducers) consider health to be an important food choice motivator [20,21]. However, as this is the first United Kingdom–based study exploring food group contributors to meat intake across consumer levels, these speculations warrant further investigation.

High consumers ate the majority of their RPM at dinner, further indicative of traditional meals that are centered on meat. Low consumers, distributed their intake more across lunch and dinner, which may imply a more flexible approach to incorporating meat into various meal contexts. All consumer groups consumed the most amount of meat on Sundays, which is in line with previous United Kingdom–based studies [22,23]. Among high consumers, RPM intake was second highest on Mondays, which is in line with the result of National Diet and Nutrition Survey analysis, which found meat intake to be higher on Mondays than any other weekday [22].

Scotland was one of the first countries to declare a formal climate emergency, in 2019, setting an ambitious net zero target of 2045. Although Scotland has halved its greenhouse gas emissions over the last 30 y, agriculture (especially livestock) is now one of the top 3 sources of Scottish greenhouse gas emissions, surpassing energy supply since 2016 [24]. These insights into RPM consumption behaviors could inform messaging strategies for the Scottish Government following their partial acceptance of the CCC’s meat reduction recommendation on the pathway to net zero [9]. Instead of simply advocating for meat reduction, these findings could support the provision of actionable steps to cut down meat intake effectively. Strategies focusing on modifying traditional meat-centric dishes and sandwiches could be impactful. Encouraging individuals to reduce meat portion sizes at dinner could also be a viable strategy. Policymakers working with the food industry to establish portion size guidelines for meat within composite main meals (e.g., ready meals) might also be effective, considering recent declines in United Kingdom meat consumption have primarily stemmed from portion size reductions [25]. Sandwiches contribute significantly to RPM intake, representing 15% among high consumers and 13% of total meat intake in Scotland (notably ham sandwiches) [13]. However, there is a notable disparity in the availability of meat and meat-free sandwich options in United Kingdom food retailers, with 59% containing meat—38% containing RPM—and only 12% being meat-free [26]. Increasing the relative availability of meat-free sandwiches could be an effective strategy for reducing meat selection, as evidenced by previous research that has increased the availability of meat-free meals [27,28]. Moreover, this could support a shift in meat consumption social norms and inspire individuals to explore and create meat-free sandwich fillings at home. These findings are particularly timely in light of the recently published draft national Good Food Nation Plan in Scotland, which provides an overarching framework for future Scottish food policy that will be taken forward at both national and local levels [29]. The national plan includes 6 outcomes, many relating to RPM production and consumption, such as health and nutrition, sustainability and animal welfare, economic and social wellbeing, and food cultures.

A major strength of this analysis is the use of contemporary dietary intake data from a nationally representative survey. Further, our use of disaggregated meat data allowed us to estimate RPM intake from mixed dishes, providing valuable insights into the varying contributions of food groups across consumer levels. However, underreporting—an inherent limitation of self-reported dietary assessment methods [30,31]—may have varied across consumer groups. Previous research has suggested higher underreporting among males in high-income countries [32] and individuals living in lower socioeconomic status areas [33]. As we identified high RPM consumers were more likely to be male and living in the most deprived areas, it is plausible they underreported to a greater extent than low consumers. Additionally, portion size reporting errors may have varied by dish type. Mixed dishes (e.g., chili con carne) may have higher discrepancies when shared among household members than discrete food items (e.g., a beef burger). Nevertheless, our primary focus was on contextualizing RPM consumption behaviors rather than establishing absolute intake levels. Further, because purchase locations were reported at the meal level rather than the ingredient level and because of variability in participants’ meal reporting, we could not establish the exact RPM items purchased from supermarkets (e.g., raw ingredients compared with prepared meals). This merits further investigation in future research. This analysis presents a detailed understanding of RPM consumption behaviors among low, medium, and high adult consumers in Scotland. Understanding the distinct consumption behaviors, of high consumers in particular, could be used to inform the design of effective strategies and policies aligned with meat reduction targets.

## Author contributions

The authors’ responsibilities were as follows – CS, RR, GM, FC, AM, LMJ: designed research; CS: conducted research and analyzed data; LMJ: directly accessed and verified underlying data; CS: wrote the first draft of the manuscript; LMJ, CS: had primary responsibility for the final content; and all authors: critically reviewed and approved the final manuscript.

## Conflicts of interest

The authors report no conflicts of interest.

## Funding

This research was funded by Food Standards Scotland (award number FSS/2021/012). Food Standards Scotland were involved in the study design, writing of the manuscript, and the decision to submit the manuscript for publication. At the time of this research, CS and RR were funded by Food Standards Scotland. LMJ is funded by Medical Research Council/UK Research and Innovation (Grant Ref: MR/T044527/1) and GMcN is funded by Food Standards Scotland and the University of Edinburgh.

## Data availability

Data from the Scottish Health Survey can be accessed on the UK Data Service (https://ukdataservice.ac.uk/). All analysis codes and files are publicly and freely available on GitHub: https://github.com/Cristina-Stewart/SHeS_Red-and-processed-meat-consumption.

## References

[1] Micha R., Wallace S.K., Mozaffarian D. (2010). Red and processed meat consumption and risk of incident coronary heart disease, stroke, and diabetes mellitus: a systematic review and meta-analysis. Circulation.

[2] Bouvard V., Loomis D., Guyton K.Z., Grosse Y., Ghissassi F.E., Benbrahim-Tallaa L. (2015). Carcinogenicity of consumption of red and processed meat. Lancet Oncol.

[3] Huang T., Yang B., Zheng J., Li G., Wahlqvist M.L., Li D. (2012). Cardiovascular disease mortality and cancer incidence in vegetarians: a meta-analysis and systematic review. Ann. Nutr. Metab..

[4] The Scientific Advisory Committee on Nutrition (2010).

[5] The Scottish Government (2016). Revised dietary goals for Scotland—March 2016. https://www.foodstandards.gov.scot/downloads/Scottish_Dietary_Goals_for_Scotland_-_March_2016.pdf.

[6] Poore J., Nemecek T. (2018). Reducing food’s environmental impacts through producers and consumers. Science..

[7] Committee on Climate Change (2020). Land use: Policies for a Net Zero UK [Internet]. https://www.theccc.org.uk/publication/land-use-policies-for-a-net-zero-uk.

[8] Committee on Climate Change (2022). 2022 Progress Report to Parliament. The CCC’s annual assessment of UK progress in reducing emissions. https://www.theccc.org.uk/publication/2022-progress-report-to-parliament/.

[9] The Scottish Government (2023). https://www.gov.scot/publications/scotlands-response-climate-change-committees-ccc-annual-progress-report-2022-recommendations/documents.

[10] Willett W., Rockström J., Loken B., Springmann M., Lang T., Vermeulen S. (2019). Food in the Anthropocene: the EAT–Lancet Commission on healthy diets from sustainable food systems. Lancet.

[11] Beal T., Ortenzi F., Fanzo J. (2023). Estimated micronutrient shortfalls of the EAT–Lancet planetary health diet. Lancet Planet Health.

[12] Stewart C., Piernas C., Cook B., Jebb S.A. (2021). Trends in UK meat consumption: analysis of data from years 1–11 (2008–09 to 2018–19) of the National Diet and Nutrition Survey rolling programme. Lancet Planet Health.

[13] Stewart C., McNeill G., Runions R., Comrie F., McDonald A., Jaacks L. (2023). Meat and milk product consumption in Scottish adults: Insights from a national survey. Research Square.

[14] Hinchliffe S., Wilson V., Macflarlane J., Gounari X., Roberts C. (2021). https://www.gov.scot/publications/scottish-health-survey-2021-volume-2-technical-report.

[15] Fitt E., Mak T.N., Stephen A.M., Prynne C., Roberts C., Swan G. (2010). Disaggregating composite food codes in the UK National Diet and Nutrition Survey food composition databank. Eur. J. Clin. Nutr..

[16] Bates B., Collins D., Jones K., Page P., Roberts C., Steer T. (2020). https://www.gov.uk/government/statistics/ndns-results-from-years-9-to-11-2016-to-2017-and-2018-to-2019.

[17] Clonan A., Roberts K.E., Holdsworth M. (2016). Socioeconomic and demographic drivers of red and processed meat consumption: implications for health and environmental sustainability. Proc. Nutr. Soc..

[18] Maguire E.R., Monsivais P. (2015). Socio-economic dietary inequalities in UK adults: an updated picture of key food groups and nutrients from national surveillance data. Br. J. Nutr..

[19] Barton K.L., Chambers S., Anderson A.S., Wrieden W.L. (2018). Time to address the double inequality of differences in dietary intake between Scotland and England. Br. J. Nutr..

[20] de Boer J., Schösler H., Aiking H. (2017). Towards a reduced meat diet: mindset and motivation of young vegetarians, low, medium and high meat-eaters. Appetite.

[21] Verain M.C.D., Dagevos H. (2022). Comparing meat abstainers with avid meat eaters and committed meat reducers. Front. Nutr..

[22] Horgan G.W., Scalco A., Craig T., Whybrow S., Macdiarmid J.I. (2019). Social, temporal and situational influences on meat consumption in the UK population. Appetite.

[23] Marshall D. (2005). Food as ritual, routine or convention. Consum. Mark. Cult..

[24] Scottish Government (2022). https://www.gov.scot/publications/scottish-greenhouse-gas-statistics-2020/documents/.

[25] Vonderschmidt A., Bellows A., Jaacks L.M., Alexander P., Stewart C. (2023). Contribution of meat-free days, meat-free meals, and portion sizes to UK meat consumption declines. Eur. J. Public Health [Internet].

[26] (2022). Eating Better.

[27] Pechey R., Bateman P., Cook B., Jebb S.A. (2022). Impact of increasing the relative availability of meat-free options on food selection: two natural field experiments and an online randomised trial. Int. J. Behav. Nutr. Phys. Act..

[28] Garnett E.E., Balmford A., Sandbrook C., Pilling M.A., Marteau T.M. (2019). Impact of increasing vegetarian availability on meal selection and sales in cafeterias. Proc. Natl. Acad. Sci. U.S.A..

[29] Scottish Government (2024 Jan). National Good Food Nation Plan [Internet]. https://www.gov.scot/publications/national-good-food-nation-plan/documents/.

[30] Foster E., Lee C., Imamura F., Hollidge S.E., Westgate K.L., Venables M.C. (2019). Validity and reliability of an online self-report 24-h dietary recall method (Intake24): a doubly labelled water study and repeated-measures analysis. J. Nutr. Sci..

[31] Ravelli M.N., Schoeller D.A. (2020). Traditional self-reported dietary instruments are prone to inaccuracies and new approaches are needed. Front. Nutr..

[32] McKenzie B.L., Coyle D.H., Santos J.A., Burrows T., Rosewarne E., Peters S.A. (2021). Investigating sex differences in the accuracy of dietary assessment methods to measure energy intake in adults: a systematic review and meta-analysis. Am. J. Clin. Nutr..

[33] Grech A., Hasick M., Gemming L., Rangan A. (2021). Energy misreporting is more prevalent for those of lower socio-economic status and is associated with lower reported intake of discretionary foods. Br. J. Nutr..

