# Supplementary material for: Red and Red Processed Meat Consumption Behaviors in Scottish Adults
Source: Curr Dev Nutr. 2024 May 16;8(6):103777. doi: 10.1016/j.cdnut.2024.103777 (PMC11192775; doi:10.1016/j.cdnut.2024.103777)
Supplement: Supplementary Table 1 [file mmc1.docx]

Red and Red Processed Meat Consumption Behaviours in Scottish Adults.

C Stewart et al 2024.

**Supplementary Table 1.**

Food items captured within red and red processed meat definition. Two poultry items (shaded grey) were identified in the mixed-meat disaggregated categories and removed.

| **Disaggregated meat variable** | **Food item** | **Animal type** |
| --- | --- | --- |
| Processed Red Meat | Corned beef , not canned | Beef |
|  | Corned beef hash | Beef |
|  | Corned beef, canned | Beef |
|  | Pastrami | Beef |
|  | BLT sandwich with white/malted bread | Pork |
|  | BLT sandwich with wholemeal/oatmeal bread | Pork |
|  | Bacon and cheese grills | Pork |
|  | Bacon and egg in a bun/muffin (e.g. bacon and egg McMuffin) | Pork |
|  | Bacon cheese burger, in a bun | Pork |
|  | Bacon cheeseburger, including bun | Pork |
|  | Bacon sandwich with mayo wholemeal/oatmeal bread | Pork |
|  | Bacon sandwich with white/malted bread | Pork |
|  | Bacon sandwich with wholemeal/oatmeal bread | Pork |
|  | Bacon streaky, smoked, grilled | Pork |
|  | Bacon, back/middle, smoked, grilled (including fat) | Pork |
|  | Bacon, back/middle, smoked, grilled (fat removed) | Pork |
|  | Bacon, back/middle, unsmoked, grilled (including fat) | Pork |
|  | Bacon, back/middle, unsmoked, grilled (fat removed) | Pork |
|  | Bacon, smoked, fried (fat removed) | Pork |
|  | Bacon, smoked, fried (including fat) | Pork |
|  | Bacon, streaky, smoked, grilled | Pork |
|  | Bacon, streaky, unsmoked, grilled | Pork |
|  | Bacon, type and cooking method unknown | Pork |
|  | Bacon, unsmoked, fried (fat removed) | Pork |
|  | Bacon, unsmoked, fried (including fat) | Pork |
|  | Bacon/gammon joint, boiled (fat removed) | Pork |
|  | Bacon/gammon joint, boiled (including fat) | Pork |
|  | Beef bourguignon | Pork |
|  | Beef lasagne | Pork |
|  | Breakfast sandwich with white/malted bread | Pork |
|  | Breakfast sandwich with wholemeal/oatmeal bread | Pork |
|  | Breakfast sub roll/baguette | Pork |
|  | Burger King bacon cheeseburger | Pork |
|  | Carbonara pasta sauce | Pork |
|  | Charcuterie | Pork |
|  | Cheese and ham sandwich with white/malted bread | Pork |
|  | Cheese and ham sandwich with wholemeal/oatmeal bread | Pork |
|  | Chicken and bacon sandwich with mayo with wholemeal/oatmeal bread | Pork |
|  | Chicken and bacon sandwich with white/malted bread | Pork |
|  | Chicken and bacon sandwich with wholemeal/oatmeal bread | Pork |
|  | Chicken and bacon sub roll/baguette | Pork |
|  | Chicken and bacon wrap | Pork |
|  | Chicken and ham sandwich with mayo with white/malted bread | Pork |
|  | Chicken and ham sandwich with mayo with white/malted bread | Pork |
|  | Chicken mayonnaise sandwich fillers (e.g. chicken and sweetcorn, tikka, coronation) | Pork |
|  | Chicken mayonnaise sandwich fillers, low fat | Pork |
|  | Chicken with a tomato and cheese sauce | Pork |
|  | Dairylea Lunchables (includes cheese, meat and crackers) | Pork |
|  | Gammon steak, fried (fat removed) | Pork |
|  | Gammon steak, fried (including fat) | Pork |
|  | Gammon steak, smoked, grilled (fat removed) | Pork |
|  | Gammon steak, smoked, grilled (including fat) | Pork |
|  | Gammon steak, unsmoked, grilled (fat removed) | Pork |
|  | Glazed baked gammon | Pork |
|  | Ham and cheese slice/pie (with or without vegetable) | Pork |
|  | Ham and cheese sub roll/baguette | Pork |
|  | Ham and egg sub roll/baguette | Pork |
|  | Ham salad sandwich with mayo with white/malted bread | Pork |
|  | Ham salad sandwich with mayo with wholemeal/oatmeal bread | Pork |
|  | Ham salad sandwich with white/malted bread | Pork |
|  | Ham salad sandwich with wholemeal/oatmeal bread | Pork |
|  | Ham salad sub roll/baguette | Pork |
|  | Ham sandwich with mayo with white/malted bread | Pork |
|  | Ham sandwich with mayo with wholemeal/oatmeal bread | Pork |
|  | Ham sandwich with white/malted bread | Pork |
|  | Ham sandwich with wholemeal/oatmeal bread | Pork |
|  | Ham, low fat | Pork |
|  | Ham, not smoked | Pork |
|  | Ham, smoked | Pork |
|  | Ham, tinned | Pork |
|  | Loaded potato skins (e.g with cheese & bacon) | Pork |
|  | Luncheon meat, not canned | Pork |
|  | Luncheon meat/chopped pork, canned (e.g. spam) | Pork |
|  | Mcdonalds bacon cheese burger | Pork |
|  | Meat based quiche (e.g. quiche lorraine) | Pork |
|  | Meat based quiche (e.g. quiche lorraine), reduced fat | Pork |
|  | Meat feast sub roll/baguette | Pork |
|  | Meat pizza (e.g. Hawaiian, pepperoni, meat feast) | Pork |
|  | Meat pizza (e.g. Hawaiian, pepperoni, meat feast), takeaway/restaurant | Pork |
|  | Meat pizza (e.g. Hawaiian, pepperoni, meat feast), stuffed crust, takeaway/restaurant | Pork |
|  | Meat pizza (e.g. Hawaiian, pepperoni, meat feast), stuffed crust | Pork |
|  | Meat savoury pastry (e.g. ham and cheese) | Pork |
|  | Mixed pizza (e.g. chicken and bacon) | Pork |
|  | Mixed pizza (e.g. chicken and bacon), takeaway/restaurant | Pork |
|  | Mixed pizza (e.g. chicken and bacon), stuffed crust | Pork |
|  | Mixed pizza (e.g. chicken and bacon), stuffed crust, takeaway/restaurant | Pork |
|  | Omelette with fish/seafood (e.g. prawn) | Pork |
|  | Omelette with ham & cheese | Pork |
|  | Omelette with meat (e.g. ham) | Pork |
|  | Parma Ham | Pork |
|  | Pasta carbonara (e.g. spaghetti) | Pork |
|  | Pasta carbonara (e.g. spaghetti), ready meal | Pork |
|  | Pasta with meat and creamy sauce | Pork |
|  | Pate sandwich with white/malted bread | Pork |
|  | Pate sandwich with wholemeal/oatmeal bread | Pork |
|  | Pea and ham soup | Pork |
|  | Pepperami or snack salami | Pork |
|  | Pigs in blankets | Pork |
|  | Prosciutto | Pork |
|  | Ready cooked savoury rice (e.g. Uncle Bens) | Pork |
|  | Salami | Pork |
|  | Sausage sandwich with ketchup with white/malted bread or roll | Pork |
|  | Sausage sandwich with ketchup with wholemeal/oatmeal bread or roll | Pork |
|  | Scotch egg (including mini/picnic size) | Pork |
|  | Serrano ham | Pork |
| Burgers | Bacon cheese burger, in a bun | Beef |
|  | Bacon cheeseburger, including bun | Beef |
|  | Beef burger, 100% beef, grilled (no bun) | Beef |
|  | Beef burger, fried (no bun) | Beef |
|  | Beef burger, grilled (no bun) | Beef |
|  | Beef burger, reduced fat, grilled (no bun) | Beef |
|  | Beef burger/hamburger, in a bun, not quarter pounder | Beef |
|  | Beef meatballs, grilled or oven baked | Beef |
|  | Beef rissoles | Beef |
|  | Burger King Double Whooper with cheese | Beef |
|  | Burger King Whooper, no cheese | Beef |
|  | Burger King Whopper with cheese | Beef |
|  | Burger King bacon cheeseburger | Beef |
|  | Cheese burger, in a bun, not quarter pounder | Beef |
|  | Cheese burger, in a bun, quarter pounder | Beef |
|  | Cheeseburger, in a bun, quarter pounder | Beef |
|  | McDonald's Big Mac | Beef |
|  | McDonalds Cheeseburger | Beef |
|  | Mcdonalds bacon cheese burger | Beef |
|  | Rustlers burger | Beef |
|  | Hot dog/frankfurter with sauce and onions in a bun | Pork |
|  | Lamb burger (no bun) | Lamb |
| Sausages | Beef Sausage, grilled | Beef |
|  | Venison sausage | Game |
|  | Chicken/turkey sausage | Poultry |
|  | Baked beans and sausages | Pork |
|  | Bierwurst/garlic sausage | Pork |
|  | Bratwurst, grilled | Pork |
|  | Breakfast sandwich with white/malted bread | Pork |
|  | Breakfast sandwich with wholemeal/oatmeal bread | Pork |
|  | Breakfast sub roll/baguette | Pork |
|  | Chicken and prawn paella, ready meal, reduced fat | Pork |
|  | Chorizo | Pork |
|  | Hot dog/frankfurter | Pork |
|  | Hot dog/frankfurter with sauce in a bun | Pork |
|  | Meat pizza (e.g. Hawaiian, pepperoni, meat feast) | Pork |
|  | Meat pizza (e.g. Hawaiian, pepperoni, meat feast), takeaway/restaurant | Pork |
|  | Mixed meat/seafood paella | Pork |
|  | Pasta with meat and tomato-based sauce, canned (e.g. pasta with sausages) | Pork |
|  | Pigs in blankets | Pork |
|  | Polony | Pork |
|  | Pork Sausage, reduced fat | Pork |
|  | Pork sausage, grilled | Pork |
|  | Sausage and egg in a bun/muffin (e.g. Sausage and egg McMuffin) | Pork |
|  | Sausage and egg in a bun/muffin (e.g. Bacon and egg McMuffin) | Pork |
|  | Sausage casserole | Pork |
|  | Sausage in batter, fried | Pork |
|  | Sausage meat stuffing | Pork |
|  | Sausage roll | Pork |
|  | Sausage sandwich with ketchup with white/malted bread or roll | Pork |
|  | Sausage sandwich with ketchup with wholemeal/oatmeal bread or roll | Pork |
|  | Sausage, fried | Pork |
|  | Smoked Sausage | Pork |
|  | Spaghetti bolognese, canned | Pork |
|  | Square/Lorne sausage | Pork |
|  | Toad in the hole | Pork |
| Offal | Calf liver | Beef |
|  | Ox Liver | Beef |
|  | Oxtail | Beef |
|  | Oxtail soup, canned | Beef |
|  | Lambs liver, fried | Lamb |
|  | Lambs liver, stewed | Lamb |
|  | Lambs liver, stewed in gravy | Lamb |
|  | Haggis | Lamb |
|  | Black pudding | Pork |
|  | Chicken and vegetable soup, homemade | Pork |
|  | Cup a soup, made up | Pork |
|  | Pate (e.g. brussels liver pate / duck and orange pate) | Pork |
|  | Pate, canned | Pork |
|  | Pate, reduced fat | Pork |
|  | Tongue (e.g. pork, ox) | Pork |
|  | Chicken liver | Poultry |
| Other red meat | Game pie (e.g. venison, rabbit, pheasant) | Game |
|  | Meat risotto (e.g. beef/ham) | Game |
|  | Roast/stewed rabbit | Game |
|  | Venison, roasted or stewed | Game |
