# Supplementary material for: Red and Red Processed Meat Consumption Behaviors in Scottish Adults
Source: Curr Dev Nutr. 2024 May 16;8(6):103777. doi: 10.1016/j.cdnut.2024.103777 (PMC11192775; doi:10.1016/j.cdnut.2024.103777)
Supplement: Supplementary Methods [file mmc2.docx]

Red and Red Processed Meat Consumption Behaviours in Scottish Adults.

C Stewart et al 2024.

**Supplementary Methods - Categorisation of meal occasion and purchase location**

Meal occasion

Respondents were asked to assign a ‘meal name’ to each meal they reported in their recalls. They could select from one of the following nine multiple-choice options:

1. Early snack or drink
2. Breakfast
3. Morning snack or drink
4. Lunch
5. Afternoon snack or drink
6. Dinner
7. Evening meal
8. Late snack or drink
9. Other (asked to specify)

The timings of all meal occasions varied considerably between respondents (**Table S1**). Notably, as the time of the individual snack and drink items (i.e. early, morning, afternoon and late) were not mutually exclusive, we grouped them together (renaming them snacks, which is more relevant for this analysis). Further, we combined ‘evening meal’ and ‘dinner’ together.

**Table S1.** Range of times pre-specified meal names were reported in SHeS 2021

| **Meal Name** | **Range of times reported** |
| --- | --- |
| Early snack or drink | 02:00-22:00 |
| Breakfast | 01:00-22:30 |
| Morning snack or drink | 01:30-16:30 |
| Lunch | 02:30-20:00 |
| Afternoon snack or drink | 03:30-22:00 |
| Dinner | 16:30-22:30 |
| Evening meal | 00:00-23:45 |
| Late snack or drink | 00:00-23:45 |

We manually reviewed the free-text meal names provided when a responded selected ‘other’, and re-coded to existing categories where appropriate. We identified the below inconsistencies and to rectify them, reviewed each meal in the context of the participant’s recall, and other recalls where appropriate, to improve the accuracy of the reported meal occasion:

1. Some respondents provided two different meal names within the same mealtime – re-coded to ensure the same mealtime had only one name.
   1. For example, one respondent listed various items at 18:00, all labelled as ‘evening meal’, including new potatoes, red wine sauce and asparagus, except for chicken legs, which were labelled as ‘chicken’. Here, we renamed ‘chicken’ to ‘evening meal’.
2. Some respondents named the entire meal after the items (this was particularly true for snack items) – recoded to ensure meal names matched the pre-specified multiple-choice options.
   1. For example, one respondent named their meal “Cottage cheese and ham on Ryvita”.
   2. Here, we re-coded this meal to ‘lunch’ as the respondent hadn’t reported lunch on this day (but did so on their subsequent recall) and consumed this meal at 16:30, between their afternoon snack (14:00) and evening meal (19:00).

Purchase location

For each meal reported, respondents were asked where they purchased the meal or its ingredients. They could select from one of the following eleven multiple-choice options:

1. Supermarket / local shop / petrol station - household shopping
2. Supermarket / local shop / petrol station - food on the go
3. Burger, chip or kebab van / 'street food'
4. Cafe / coffee shop / sandwich bar / deli
5. Canteen at work or school / university / college
6. Fast food / take-away outlet
7. Food bank (charity/community) or government food delivery scheme (food boxes/parcels)
8. Leisure centre / recreation or entertainment venue
9. Restaurant or pub
10. Don't know
11. Other (asked to specify)

A total of 108 meals containing red and red processed meat (2%) were purchased from ‘other’ locations. We manually reviewed these responses and re-categorised 15 into existing multiple-choice options:

- ‘hotel’ (n=12) into ‘Restaurant or pub’
- ‘McDonald’s’ and ‘Greggs’ (total n=3) into ‘Fast food / take-away outlet’.

All remaining ‘other’ locations, such as butchers, homemade/homegrown, and recipe subscription boxes, were analysed together.

For analyses, we further collapsed the above eleven multiple-choice options into: 1) Supermarkets; 2) Cafés, restaurants & takeaways; and 3) other:

**Supermarkets**

1. Supermarket / local shop / petrol station - household shopping
2. Supermarket / local shop / petrol station - food on the go

**Cafés, restaurants & takeaways**

1. Burger, chip or kebab van / 'street food'
2. Cafe / coffee shop / sandwich bar / deli
3. Fast food / take-away outlet
4. Restaurant or pub

**Other**

1. Canteen at work or school / university / college
2. Food bank (charity/community) or government food delivery scheme (food boxes/parcels)
3. Leisure centre / recreation or entertainment venue
4. Don't know
5. Other (asked to specify)
