# Supplementary material for: Red and Red Processed Meat Consumption Behaviors in Scottish Adults
Source: Curr Dev Nutr. 2024 May 16;8(6):103777. doi: 10.1016/j.cdnut.2024.103777 (PMC11192775; doi:10.1016/j.cdnut.2024.103777)
Supplement: Supplementary Data [file mmc3.docx]

Red and Red Processed Meat Consumption Behaviours in Scottish Adults.

C Stewart et al 2024.

**Supplementary Data**

Contents

[**Table 1.** Demographic characteristics 2](#_Toc165979942)

[**Table 2**. Mean intake (g and % contribution) of processed and unprocessed red meat 3](#_Toc165979943)

[**Table 3.** Percent contribution of animal types to processed red meat intake 4](#_Toc165979944)

[**Table 4**. Mean intake (g and % contribution) of RPM by meal occasion 5](#_Toc165979945)

[**Table 5**. Mean intake (g) of RPM by day of the week 6](#_Toc165979946)

[**Table 6.** Mean intake (g and % contribution) of RPM by purchase location 7](#_Toc165979947)

[**Table 7.** Mean intake of RPM (g and % contribution) from food categories 8](#_Toc165979948)

[**Table 8.** Mean intake of RPM (g and % contribution) from main food groups 9](#_Toc165979949)

[**Table 9.** Mean intake of RPM (g and % contribution) from sub food groups 11](#_Toc165979950)

[**Table 10.** Consumption frequency of RPM items among low consumers 13](#_Toc165979951)

[**Table 10.** Consumption frequency of RPM items among medium consumers 15](#_Toc165979952)

[**Table 11.** Consumption frequency of RPM items among high consumers 17](#_Toc165979953)

# **Table 1.** Demographic characteristics

|  | **Non consumers** (0 g/d), *n=953* | | **All consumers** (>0g/d), *n=2494* | | **Low consumers** (>0 to ≤34.5 g/d), *n=824* | | **Medium consumers** (>34.5 to 70 g/d), *n=784* | | **High consumers** (>70 g/d), *n=886* | |
| --- | --- | --- | --- | --- | --- | --- | --- | --- | --- | --- |
|  | Unweighted n | Weighted  % | Unweighted  n | Weighted  % | Unweighted  n | Weighted  % | Unweighted  n | Weighted  % | Unweighted  n | Weighted  % |
| **Gender** |  |  |  |  |  |  |  |  |  |  |
| Male | 330 | 41.8 | 1,083 | 50.7 | 276 | 37.9 | 339 | 51.2 | 468 | 60.2 |
| Female | 623 | 58.2 | 1,411 | 49.3 | 548 | 62.1 | 445 | 48.8 | 418 | 39.8 |
| **Age group (years)** |  |  |  |  |  |  |  |  |  |  |
| 16-34 | 168 | 30.7 | 402 | 27.2 | 122 | 24.2 | 131 | 28.3 | 149 | 28.4 |
| 35-54 | 322 | 32.7 | 769 | 31.7 | 232 | 30.2 | 245 | 29.6 | 292 | 34.6 |
| 55-74 | 392 | 27.7 | 1,099 | 30.9 | 399 | 35.6 | 326 | 30.1 | 374 | 27.8 |
| ≥75 | 71 | 8.9 | 224 | 10.4 | 71 | 10.0 | 82 | 12.0 | 71 | 9.2 |
| **Ethnicity** |  |  |  |  |  |  |  |  |  |  |
| White: Scottish | 629 | 65.5 | 1,861 | 76.0 | 611 | 75.0 | 571 | 75.4 | 679 | 77.4 |
| White: Other British | 217 | 18.4 | 434 | 14.8 | 142 | 15.3 | 146 | 15.5 | 146 | 13.8 |
| White: Other | 52 | 6.0 | 134 | 5.4 | 51 | 5.8 | 46 | 5.9 | 37 | 4.6 |
| Asian | 28 | 6.3 | 34 | 2.2 | 11 | 2.3 | 9 | 1.7 | 14 | 2.5 |
| Other minority ethnic | 24 | 3.5 | 31 | 1.6 | 9 | 1.5 | 12 | 1.5 | 10 | 1.8 |
| Refused | 2 | 0.2 | 0 | 0.0 | 0 | 0.0 | 0 | 0.0 | 0 | 0.0 |
| Don't know | 1 | 0.1 | 0 | 0.0 | 0 | 0.0 | 0 | 0.0 | 0 | 0.0 |
| **SIMD quintile** |  |  |  |  |  |  |  |  |  |  |
| 1 (Most deprived) | 122 | 19.5 | 303 | 18.1 | 85 | 15.9 | 91 | 16.7 | 127 | 21.1 |
| 2 | 168 | 20.1 | 428 | 19.8 | 129 | 19.1 | 139 | 20.1 | 160 | 20.2 |
| 3 | 189 | 16.9 | 519 | 19.7 | 187 | 20.6 | 153 | 20.0 | 179 | 18.6 |
| 4 | 240 | 21.2 | 659 | 21.2 | 228 | 21.7 | 191 | 19.6 | 240 | 22.4 |
| 5 (Least deprived) | 234 | 22.3 | 585 | 21.3 | 195 | 22.7 | 210 | 23.7 | 180 | 17.7 |

| Data are from adults (≥16 years) in the 2021 Scottish Health Survey.  Abbreviations: RPM; Red and red processed meat, SIMD; Scottish Index of Multiple Deprivation |
| --- |

# **Table 2**. Mean intake (g and % contribution) of processed and unprocessed red meat

|  | **Intake (grams)** | | | | | | **% contribution** | |
| --- | --- | --- | --- | --- | --- | --- | --- | --- |
|  | N | Mean | Median | SD | 2.5th percentile | 97.5th percentile | Mean | SD |
| **All consumers** |  |  |  |  |  |  |  |  |
| Total red and red processed meat | 2494 | 66.1 | 54.3 | 50.8 | 4.8 | 195.5 | - | - |
| Processed red meat | 2494 | 36.1 | 25.5 | 40.9 | 0.0 | 130.8 | 56.1 | 41.9 |
| Unprocessed red meat | 2494 | 30.0 | 16.7 | 39.4 | 0.0 | 141.4 | 43.9 | 41.9 |
| **Low consumers** |  |  |  |  |  |  |  |  |
| Total red and red processed meat | 824 | 19.2 | 20.7 | 9.9 | 0.6 | 34.0 | - | - |
| Processed red meat | 824 | 11.0 | 8.0 | 11.7 | 0.0 | 33.0 | 57.2 | 49.7 |
| Unprocessed red meat | 824 | 8.2 | 0.1 | 11.5 | 0.0 | 31.2 | 42.8 | 49.7 |
| **Medium consumers** |  |  |  |  |  |  |  |  |
| Total red and red processed meat | 784 | 50.0 | 20.7 | 9.7 | 0.6 | 34.0 | - | - |
| Processed red meat | 784 | 28.5 | 8.0 | 20.6 | 0.0 | 33.0 | 57.7 | 40.9 |
| Unprocessed red meat | 784 | 21.5 | 0.1 | 21.2 | 0.0 | 31.2 | 42.3 | 40.9 |
| **High consumers** |  |  |  |  |  |  |  |  |
| Total red and red processed meat | 886 | 116.8 | 101.0 | 45.7 | 71.3 | 255.0 | - | - |
| Processed red meat | 886 | 62.4 | 59.9 | 50.7 | 0.0 | 189.0 | 53.8 | 36.4 |
| Unprocessed red meat | 886 | 54.5 | 45.0 | 49.4 | 0.0 | 172.8 | 46.2 | 36.4 |

Data are from adults (≥16 years) in the 2021 Scottish Health Survey.

Consumers (>0g total red and red processed meat); low consumers (>0g to ≤34.5g/d); medium consumers (>34.5g to 70g/d); high consumers (>70g/d).

Abbreviations: RPM; Red and red processed meat, SD; Standard deviation

# **Table 3.** Percent contribution of animal types to processed red meat intake

|  |  | **% contribution** | |
| --- | --- | --- | --- |
|  | N | Mean | SD |
| **All consumers** |  |  |  |
| Beef | 2494 | 13.2 | 30.2 |
| Pork | 2494 | 86.5 | 30.5 |
| Lamb | 2494 | 0.1 | 3.7 |
| Game | 2494 | 0.1 | 3.3 |
| **Low consumers** |  |  |  |
| Beef | 824 | 7.8 | 26.0 |
| Pork | 824 | 91.9 | 26.5 |
| Lamb | 824 | 0.1 | 2.3 |
| Game | 824 | 0.2 | 4.9 |
| **Medium consumers** |  |  |  |
| Beef | 784 | 18.2 | 35.8 |
| Pork | 784 | 81.5 | 36.1 |
| Lamb | 784 | 0.3 | 5.1 |
| Game | 784 | 0.1 | 2.3 |
| **High consumers** |  |  |  |
| Beef | 886 | 12.6 | 27.9 |
| Pork | 886 | 87.2 | 28.1 |
| Lamb | 886 | 0.1 | 3.1 |
| Game | 886 | 0.1 | 2.8 |

Data are from adults (≥16 years) in the 2021 Scottish Health Survey.

Consumers (>0g total red and red processed meat); low consumers (>0g to ≤34.5g/d); medium consumers (>34.5g to 70g/d); high consumers (>70g/d).

Further details on the categorisation of animal type can be found in Supplementary Table 1.

Abbreviations: RPM; Red and red processed meat, SD; Standard deviation

# **Table 4**. Mean intake (g and % contribution) of RPM by meal occasion

|  |  | **Intake (grams)** | | **% contribution** | |
| --- | --- | --- | --- | --- | --- |
|  | N | Mean | SD | Mean | SD |
| **All consumers** |  |  |  |  |  |
| Breakfast | 2494 | 5.8 | 18.6 | 7.5 | 21.3 |
| Lunch | 2494 | 20.0 | 27.6 | 34.0 | 38.9 |
| Dinner | 2494 | 35.8 | 40.2 | 52.0 | 41.4 |
| Snacks | 2494 | 4.6 | 16.4 | 6.6 | 20.8 |
| **Low consumers** |  |  |  |  |  |
| Breakfast | 824 | 1.0 | 4.7 | 5.1 | 22.7 |
| Lunch | 824 | 7.4 | 10.7 | 40.2 | 49.3 |
| Dinner | 824 | 9.7 | 11.8 | 48.0 | 50.5 |
| Snacks | 824 | 1.2 | 5.2 | 6.7 | 25.7 |
| **Medium consumers** |  |  |  |  |  |
| Breakfast | 784 | 3.5 | 10.0 | 7.1 | 20.8 |
| Lunch | 784 | 17.5 | 19.0 | 35.3 | 38.3 |
| Dinner | 784 | 26.1 | 21.2 | 51.8 | 41.0 |
| Snacks | 784 | 2.9 | 9.9 | 5.8 | 19.5 |
| **High consumers** |  |  |  |  |  |
| Breakfast | 886 | 11.7 | 26.6 | 9.6 | 20.4 |
| Lunch | 886 | 31.9 | 35.5 | 28.0 | 29.2 |
| Dinner | 886 | 64.6 | 47.1 | 55.2 | 33.8 |
| Snacks | 886 | 8.6 | 23.3 | 7.1 | 18.0 |

Data are from adults (≥16 years) in the 2021 Scottish Health Survey.

Consumers (>0g total red and red processed meat); low consumers (>0g to ≤34.5g/d); medium consumers (>34.5g to 70g/d); high consumers (>70g/d).

Further details on the categorisation of meal occasion can be found in Supplementary Methods.

RPM; Red and red processed meat, SD; Standard deviation

# **Table 5**. Mean intake (g) of RPM by day of the week

|  |  | **Intake (grams)** | |
| --- | --- | --- | --- |
|  | N | Mean | SD |
| **All consumers** |  |  |  |
| Monday | 845 | 70.7 | 69.4 |
| Tuesday | 809 | 65.9 | 69.2 |
| Wednesday | 751 | 58.4 | 72.4 |
| Thursday | 711 | 66.9 | 66.7 |
| Friday | 599 | 59.5 | 69.2 |
| Saturday | 472 | 60.4 | 62.6 |
| Sunday | 494 | 73.3 | 70.3 |
| **Low consumers** |  |  |  |
| Monday | 271 | 19.7 | 22.2 |
| Tuesday | 267 | 20.6 | 22.8 |
| Wednesday | 258 | 15.9 | 20.5 |
| Thursday | 225 | 20.6 | 24.1 |
| Friday | 217 | 20.0 | 24.4 |
| Saturday | 166 | 17.5 | 23.2 |
| Sunday | 162 | 21.4 | 22.2 |
| **Medium consumers** |  |  |  |
| Monday | 261 | 52.8 | 34.3 |
| Tuesday | 251 | 51.6 | 36.1 |
| Wednesday | 245 | 45.3 | 35.4 |
| Thursday | 224 | 54.4 | 41.2 |
| Friday | 182 | 42.6 | 36.7 |
| Saturday | 151 | 48.6 | 35.0 |
| Sunday | 157 | 54.9 | 36.4 |
| **High consumers** |  |  |  |
| Monday | 313 | 126.3 | 77.4 |
| Tuesday | 291 | 109.7 | 79.7 |
| Wednesday | 248 | 109.1 | 91.5 |
| Thursday | 262 | 109.9 | 75.0 |
| Friday | 200 | 111.6 | 84.2 |
| Saturday | 155 | 108.9 | 70.3 |
| Sunday | 175 | 131.2 | 75.8 |

Data are from adults (≥16 years) in the 2021 Scottish Health Survey.

Consumers (>0g total red and red processed meat); low consumers (>0g to ≤34.5g/d); medium consumers (>34.5g to 70g/d); high consumers (>70g/d).

Abbreviations: RPM; Red and red processed meat, SD; Standard deviation

# **Table 6.** Mean intake (g and % contribution) of RPM by purchase location

|  |  | **Intake (grams)** | | **% contribution** | |
| --- | --- | --- | --- | --- | --- |
|  | N | Mean | SD | Mean | SD |
| **All consumers** |  |  |  |  |  |
| Supermarkets | 2494 | 57.1 | 49.7 | 86.7 | 29.8 |
| Cafes, restaurants, pubs and takeaways | 2494 | 7.1 | 20.8 | 10.7 | 26.8 |
| Other | 2494 | 2.0 | 13.2 | 2.6 | 14.0 |
| **Low consumers** |  |  |  |  |  |
| Supermarkets | 824 | 17.0 | 11.1 | 88.0 | 33.2 |
| Cafes, restaurants, pubs and takeaways | 824 | 1.9 | 6.5 | 10.0 | 30.7 |
| Other | 824 | 0.3 | 2.4 | 1.9 | 14.3 |
| **Medium consumers** |  |  |  |  |  |
| Supermarkets | 784 | 43.9 | 16.1 | 87.6 | 27.5 |
| Cafes, restaurants, pubs and takeaways | 784 | 5.1 | 12.6 | 10.4 | 25.3 |
| Other | 784 | 1.0 | 5.8 | 2.0 | 12.0 |
| **High consumers** |  |  |  |  |  |
| Supermarkets | 886 | 99.8 | 53.2 | 84.9 | 28.9 |
| Cafes, restaurants, pubs and takeaways | 886 | 12.8 | 29.4 | 11.6 | 25.2 |
| Other | 886 | 4.2 | 19.8 | 3.6 | 15.1 |

Data are from adults (≥16 years) in the 2021 Scottish Health Survey.

Consumers (>0g total red and red processed meat); low consumers (>0g to ≤34.5g/d); medium consumers (>34.5g to 70g/d); high consumers (>70g/d).

Further details on the categorisation of purchase location can be found in Supplementary Methods.

Abbreviations: RPM; Red and red processed meat, SD; Standard deviation

# **Table 7.** Mean intake of RPM (g and % contribution) from food categories

|  |  | **Intake (grams)** | | **% contribution** | |
| --- | --- | --- | --- | --- | --- |
|  | N | Mean | SD | Mean | SD |
| **All consumers** |  |  |  |  |  |
| Cereals and cereal products | 2494 | 2.0 | 9.1 | 4.6 | 18.1 |
| Eggs and egg dishes | 2494 | 0.4 | 2.5 | 1.2 | 9.3 |
| Meat and meat products | 2494 | 53.2 | 49.2 | 76.2 | 36.2 |
| Sandwiches | 2494 | 10.2 | 22.2 | 15.4 | 30.1 |
| Miscellaneous | 2494 | 0.2 | 1.7 | 2.2 | 13.5 |
| **Low consumers** |  |  |  |  |  |
| Cereals and cereal products | 824 | 1.0 | 4.2 | 7.4 | 26.4 |
| Milk and milk products | 824 | 0.0 | 0.7 | 0.6 | 8.2 |
| Eggs and egg dishes | 824 | 0.5 | 3.4 | 3.0 | 17.0 |
| Meat and meat products | 824 | 14.5 | 12.1 | 69.4 | 46.7 |
| Sandwiches | 824 | 2.8 | 7.6 | 12.3 | 33.2 |
| Vegetables, potatoes | 824 | 0.1 | 1.1 | 0.6 | 7.5 |
| Miscellaneous | 824 | 0.3 | 1.2 | 6.6 | 25.4 |
| **Medium consumers** |  |  |  |  |  |
| Cereals and cereal products | 784 | 1.9 | 6.7 | 4.0 | 14.8 |
| Eggs and egg dishes | 784 | 0.3 | 2.2 | 0.7 | 5.0 |
| Meat and meat products | 784 | 38.7 | 19.3 | 76.6 | 34.8 |
| Sandwiches | 784 | 8.9 | 15.7 | 18.2 | 32.6 |
| **High consumers** |  |  |  |  |  |
| Cereals and cereal products | 886 | 2.9 | 12.4 | 3.0 | 13.0 |
| Meat and meat products | 886 | 95.9 | 51.6 | 81.1 | 27.4 |
| Sandwiches | 886 | 17.3 | 29.9 | 15.3 | 25.0 |

Data are from adults (≥16 years) in the 2021 Scottish Health Survey.

Food categories contributing <0.5% are not shown.

Food groups are defined by the UK National Diet and Nutrition Survey^1^

Consumers (>0g total red and red processed meat); low consumers (>0g to ≤34.5g/d); medium consumers (>34.5g to 70g/d); high consumers (>70g/d).

Abbreviations: RPM; Red and red processed meat, SD; Standard deviation

^1^Bates B et al. Appendix R Main and subsidiary food groups and disaggregation categories [Internet]. Public Health England; 2020 [cited 2023 Jun 19]. Available from: https://www.gov.uk/government/statistics/ndns-results-from-years-9-to-11-2016-to-2017-and-2018-to-2019

# **Table 8.** Mean intake of RPM (g and % contribution) from main food groups

|  |  | **Intake (grams)** | | **% contribution** | |
| --- | --- | --- | --- | --- | --- |
|  | N | Mean | SD | Mean | SD |
| **All consumers** |  |  |  |  |  |
| Pasta rice and other cereals | 2494 | 2.0 | 9.1 | 4.6 | 18.1 |
| Eggs and egg dishes | 2494 | 0.4 | 2.5 | 1.2 | 9.3 |
| Bacon and ham | 2494 | 8.4 | 21.7 | 14.2 | 30.0 |
| Beef veal and dishes | 2494 | 17.1 | 30.7 | 23.9 | 36.6 |
| Lamb and dishes | 2494 | 2.3 | 12.2 | 3.3 | 15.6 |
| Pork and dishes | 2494 | 4.5 | 18.8 | 4.8 | 18.1 |
| Liver & dishes | 2494 | 0.6 | 5.6 | 1.3 | 9.6 |
| Burgers and kebabs | 2494 | 4.9 | 15.9 | 7.4 | 22.2 |
| Sausages | 2494 | 8.4 | 23.9 | 9.8 | 24.6 |
| Meat pies and pastries | 2494 | 3.6 | 11.7 | 6.6 | 21.1 |
| Other meat and meat products | 2494 | 3.3 | 16.5 | 4.7 | 17.6 |
| Miscellaneous | 2494 | 0.2 | 1.7 | 2.2 | 13.5 |
| Sandwiches | 2494 | 10.2 | 22.2 | 15.4 | 30.1 |
| **Low consumers** |  |  |  |  |  |
| Pasta rice and other cereals | 824 | 1.0 | 4.2 | 7.4 | 26.4 |
| Cheese | 824 | 0.0 | 0.7 | 0.6 | 8.2 |
| Eggs and egg dishes | 824 | 0.5 | 3.4 | 3.0 | 17.0 |
| Bacon and ham | 824 | 3.7 | 8.2 | 19.3 | 39.8 |
| Beef veal and dishes | 824 | 3.8 | 9.2 | 16.7 | 38.3 |
| Lamb and dishes | 824 | 0.6 | 4.1 | 2.7 | 16.3 |
| Pork and dishes | 824 | 0.6 | 3.6 | 2.7 | 16.0 |
| Liver & dishes | 824 | 0.2 | 1.9 | 2.0 | 14.5 |
| Burgers and kebabs | 824 | 1.1 | 5.2 | 4.4 | 20.8 |
| Sausages | 824 | 1.0 | 4.7 | 5.0 | 21.9 |
| Meat pies and pastries | 824 | 2.2 | 7.1 | 9.8 | 30.6 |
| Other meat and meat products | 824 | 1.3 | 5.3 | 6.4 | 24.2 |
| Miscellaneous | 824 | 0.3 | 1.2 | 6.6 | 25.4 |
| Sandwiches | 824 | 2.8 | 7.6 | 12.3 | 33.2 |
| **Medium consumers** |  |  |  |  |  |
| Pasta rice and other cereals | 784 | 1.9 | 6.7 | 4.0 | 14.8 |
| Eggs and egg dishes | 784 | 0.3 | 2.2 | 0.7 | 5.0 |
| Bacon and ham | 784 | 6.2 | 13.7 | 12.3 | 27.5 |
| Beef veal and dishes | 784 | 14.0 | 19.4 | 27.8 | 38.1 |
| Lamb and dishes | 784 | 1.5 | 7.2 | 3.1 | 14.9 |
| Pork and dishes | 784 | 1.8 | 8.6 | 3.3 | 15.3 |
| Liver & dishes | 784 | 0.4 | 3.1 | 0.8 | 6.4 |
| Burgers and kebabs | 784 | 5.1 | 12.6 | 10.4 | 26.0 |
| Sausages | 784 | 4.8 | 12.3 | 9.2 | 23.7 |
| Meat pies and pastries | 784 | 2.8 | 9.5 | 5.5 | 18.4 |
| Other meat and meat products | 784 | 2.1 | 8.1 | 4.2 | 15.6 |
| Sandwiches | 784 | 8.9 | 15.7 | 18.2 | 32.6 |
| **High consumers** |  |  |  |  |  |
| Pasta rice and other cereals | 886 | 2.9 | 12.4 | 3.0 | 13.0 |
| Bacon and ham | 886 | 14.1 | 30.3 | 11.9 | 23.0 |
| Beef veal and dishes | 886 | 30.1 | 41.1 | 26.2 | 33.1 |
| Lamb and dishes | 886 | 4.4 | 17.6 | 4.0 | 15.6 |
| Pork and dishes | 886 | 9.8 | 27.6 | 7.8 | 20.9 |
| Liver & dishes | 886 | 1.1 | 8.2 | 1.1 | 7.5 |
| Burgers and kebabs | 886 | 7.8 | 21.4 | 7.1 | 18.9 |
| Sausages | 886 | 17.2 | 34.1 | 14.1 | 26.1 |
| Meat pies and pastries | 886 | 5.5 | 15.0 | 5.0 | 13.8 |
| Other meat and meat products | 886 | 5.9 | 24.3 | 3.9 | 13.2 |
| Sandwiches | 886 | 17.3 | 29.9 | 15.3 | 25.0 |

Data are from adults (≥16 years) in the 2021 Scottish Health Survey.

Main food groups contributing <0.5% are not shown.

Food groups are defined by the UK National Diet and Nutrition Survey^1^

Consumers (>0g total red and red processed meat); low consumers (>0g to ≤34.5g/d); medium consumers (>34.5g to 70g/d); high consumers (>70g/d).

Abbreviations: RPM; Red and red processed meat, SD; Standard deviation

^1^Bates B et al. Appendix R Main and subsidiary food groups and disaggregation categories [Internet]. Public Health England; 2020 [cited 2023 Jun 19]. Available from: https://www.gov.uk/government/statistics/ndns-results-from-years-9-to-11-2016-to-2017-and-2018-to-2019

# **Table 9.** Mean intake of RPM (g and % contribution) from sub food groups

|  |  | **Intake (grams)** | | **% contribution** | |
| --- | --- | --- | --- | --- | --- |
|  | N | Mean | SD | Mean | SD |
| **Overall** |  |  |  |  |  |
| Manufactured egg products including ready meals | 2494 | 0.3 | 2.2 | 0.9 | 8.4 |
| Pizza | 2494 | 1.7 | 8.8 | 3.3 | 15.3 |
| Pasta manufactured products & ready meals | 2494 | 0.2 | 2.1 | 0.5 | 6.6 |
| Other pasta including homemade dishes | 2494 | 0.2 | 1.2 | 0.7 | 7.4 |
| Other bacon and ham including homemade dishes | 2494 | 8.4 | 21.7 | 14.2 | 30.0 |
| Manufactured beef products including ready meals | 2494 | 0.8 | 5.1 | 1.8 | 11.7 |
| Other beef & veal including homemade recipe dishes | 2494 | 16.3 | 30.5 | 22.1 | 35.6 |
| Other lamb including homemade recipe dishes | 2494 | 2.2 | 12.0 | 3.1 | 15.2 |
| Other pork including homemade recipe dishes | 2494 | 4.2 | 18.5 | 4.5 | 17.6 |
| Liver and dishes | 2494 | 0.6 | 5.6 | 1.3 | 9.6 |
| Burgers and kebabs purchased | 2494 | 4.9 | 15.9 | 7.4 | 22.2 |
| Other sausages including homemade dishes | 2494 | 8.3 | 23.7 | 9.7 | 24.4 |
| Manufactured meat pies and pastries | 2494 | 3.6 | 11.6 | 6.5 | 21.0 |
| Other meat products manufactured incl ready meals | 2494 | 2.6 | 15.0 | 3.7 | 15.6 |
| Other meat including homemade recipe dishes | 2494 | 0.8 | 7.1 | 1.0 | 8.6 |
| Soup manufactured/ retail | 2494 | 0.1 | 0.6 | 1.1 | 9.9 |
| Soup homemade | 2494 | 0.1 | 1.5 | 0.8 | 7.6 |
| Sandwiches | 2494 | 10.2 | 22.2 | 15.4 | 30.1 |
| **Low consumers** |  |  |  |  |  |
| Other cheese | 824 | 0.0 | 0.7 | 0.6 | 8.2 |
| Manufactured egg products including ready meals | 824 | 0.5 | 3.2 | 2.4 | 15.3 |
| Other eggs and egg dishes including homemade | 824 | 0.1 | 1.0 | 0.5 | 7.4 |
| Pizza | 824 | 0.6 | 3.6 | 4.0 | 19.8 |
| Pasta manufactured products & ready meals | 824 | 0.2 | 1.8 | 1.3 | 11.0 |
| Other pasta including homemade dishes | 824 | 0.2 | 1.4 | 1.9 | 14.1 |
| Other bacon and ham including homemade dishes | 824 | 3.7 | 8.2 | 19.3 | 39.8 |
| Manufactured beef products including ready meals | 824 | 0.6 | 3.6 | 2.9 | 17.1 |
| Other beef & veal including homemade recipe dishes | 824 | 3.3 | 8.6 | 13.8 | 35.3 |
| Other lamb including homemade recipe dishes | 824 | 0.6 | 4.0 | 2.5 | 15.7 |
| Other pork including homemade recipe dishes | 824 | 0.6 | 3.6 | 2.7 | 16.0 |
| Liver and dishes | 824 | 0.2 | 1.9 | 2.0 | 14.5 |
| Burgers and kebabs purchased | 824 | 1.1 | 5.2 | 4.4 | 20.8 |
| Other sausages including homemade dishes | 824 | 1.0 | 4.5 | 4.9 | 21.5 |
| Manufactured meat pies and pastries | 824 | 2.1 | 7.1 | 9.7 | 30.5 |
| Other meat products manufactured incl ready meals | 824 | 0.9 | 4.1 | 4.8 | 21.3 |
| Other meat including homemade recipe dishes | 824 | 0.4 | 3.5 | 1.5 | 12.2 |
| Soup manufactured/ retail | 824 | 0.1 | 0.8 | 3.5 | 19.0 |
| Soup homemade | 824 | 0.1 | 0.6 | 2.1 | 14.3 |
| Savoury sauces pickles gravies & condiments | 824 | 0.1 | 0.8 | 1.0 | 10.3 |
| Sandwiches | 824 | 2.8 | 7.6 | 12.3 | 33.2 |
| **Medium consumers** |  |  |  |  |  |
| Manufactured egg products including ready meals | 784 | 0.2 | 2.0 | 0.5 | 4.6 |
| Pizza | 784 | 1.6 | 6.2 | 3.3 | 13.7 |
| Other bacon and ham including homemade dishes | 784 | 6.1 | 13.6 | 12.3 | 27.4 |
| Manufactured beef products including ready meals | 784 | 1.1 | 5.9 | 2.1 | 11.9 |
| Other beef & veal including homemade recipe dishes | 784 | 13.0 | 19.1 | 25.6 | 37.5 |
| Other lamb including homemade recipe dishes | 784 | 1.4 | 7.1 | 3.0 | 14.7 |
| Other pork including homemade recipe dishes | 784 | 1.8 | 8.5 | 3.1 | 14.8 |
| Liver and dishes | 784 | 0.4 | 3.1 | 0.8 | 6.4 |
| Burgers and kebabs purchased | 784 | 5.1 | 12.6 | 10.4 | 26.0 |
| Other sausages including homemade dishes | 784 | 4.8 | 12.3 | 9.2 | 23.7 |
| Manufactured meat pies and pastries | 784 | 2.8 | 9.5 | 5.5 | 18.4 |
| Other meat products manufactured incl ready meals | 784 | 1.7 | 7.2 | 3.4 | 13.9 |
| Other meat including homemade recipe dishes | 784 | 0.4 | 4.0 | 0.7 | 7.5 |
| Sandwiches | 784 | 8.9 | 15.7 | 18.2 | 32.6 |
| **High consumers** |  |  |  |  |  |
| Pizza | 886 | 2.7 | 12.2 | 2.8 | 12.8 |
| Other bacon and ham including homemade dishes | 886 | 14.1 | 30.3 | 11.9 | 23.0 |
| Manufactured beef products including ready meals | 886 | 0.7 | 5.3 | 0.6 | 5.0 |
| Other beef & veal including homemade recipe dishes | 886 | 29.3 | 40.8 | 25.6 | 32.9 |
| Other lamb including homemade recipe dishes | 886 | 4.1 | 17.2 | 3.8 | 15.2 |
| Manufactured pork products including ready meals | 886 | 0.6 | 5.2 | 0.7 | 6.0 |
| Other pork including homemade recipe dishes | 886 | 9.2 | 27.3 | 7.1 | 20.2 |
| Liver and dishes | 886 | 1.1 | 8.2 | 1.1 | 7.5 |
| Burgers and kebabs purchased | 886 | 7.8 | 21.4 | 7.1 | 18.9 |
| Other sausages including homemade dishes | 886 | 17.0 | 33.8 | 13.9 | 25.8 |
| Manufactured meat pies and pastries | 886 | 5.4 | 14.9 | 4.9 | 13.7 |
| Other meat products manufactured incl ready meals | 886 | 4.6 | 22.3 | 3.0 | 11.8 |
| Other meat including homemade recipe dishes | 886 | 1.3 | 10.2 | 0.9 | 6.2 |
| Sandwiches | 886 | 17.3 | 29.9 | 15.3 | 25.0 |

Data are from adults (≥16 years) in the 2021 Scottish Health Survey.

Sub food groups contributing <0.5% are not shown.

Food groups are defined by the UK National Diet and Nutrition Survey^1^

Consumers (>0g total red and red processed meat); low consumers (>0g to ≤34.5g/d); medium consumers (>34.5g to 70g/d); high consumers (>70g/d).

Abbreviations: RPM; Red and red processed meat, SD; Standard deviation

^1^Bates B et al. Appendix R Main and subsidiary food groups and disaggregation categories [Internet]. Public Health England; 2020 [cited 2023 Jun 19]. Available from: https://www.gov.uk/government/statistics/ndns-results-from-years-9-to-11-2016-to-2017-and-2018-to-2019

# **Table 10.** Consumption frequency of RPM items among low consumers

| **Food Description** | **Unweighted n** | **Weighted %** |
| --- | --- | --- |
| Ham, not smoked | 58 | 5.04% |
| Chicken and vegetable soup, homemade | 43 | 2.82% |
| Bacon, back/middle, unsmoked, grilled (fat removed) | 31 | 2.70% |
| Steak pie, slice from a large pie (including steak and kidney) | 22 | 2.67% |
| Beef lasagne | 24 | 2.66% |
| Ham, smoked | 29 | 2.64% |
| Cup a soup, made up | 26 | 2.55% |
| Meat pizza (e.g. Hawaiian, pepperoni, meat feast) | 26 | 2.29% |
| Ham sandwich with white/malted bread | 23 | 2.24% |
| Pate (e.g. brussels liver pate / duck and orange pate) | 23 | 2.20% |
| Pork sausage, grilled | 18 | 1.95% |
| Sausage roll | 16 | 1.94% |
| Cheese and ham sandwich with wholemeal/oatmeal bread | 23 | 1.88% |
| Beef burger/hamburger, in a bun, not quarter pounder | 13 | 1.87% |
| Bacon, back/middle, smoked, grilled (including fat) | 16 | 1.85% |
| Beef lasagne, ready meal | 15 | 1.85% |
| Pasta carbonara (e.g. spaghetti) | 8 | 1.58% |
| Spaghetti bolognese, home made (pasta and sauce) | 15 | 1.48% |
| Pea and ham soup | 10 | 1.39% |
| Chilli con carne | 15 | 1.27% |
| Ham, low fat | 14 | 1.25% |
| Haggis | 13 | 1.17% |
| Meat based quiche (e.g. quiche lorraine) | 14 | 1.17% |
| Sausage sandwich with ketchup with wholemeal/oatmeal bread or roll | 10 | 1.17% |
| Cheese and ham sandwich with white/malted bread | 10 | 1.09% |
| Black pudding | 12 | 1.05% |
| Serrano ham | 7 | 1.00% |
| Chicken mayonnaise sandwich fillers (e.g. chicken and sweetcorn, tikka, coronation) | 11 | 0.99% |
| Minced beef pie (including beef and potato) | 10 | 0.99% |
| Scotch egg (including mini/picnic size) | 4 | 0.97% |
| Salami | 13 | 0.96% |
| Chorizo | 13 | 0.95% |
| Mixed pizza (e.g. chicken and bacon) | 8 | 0.94% |
| Ham salad sub roll/baguette | 11 | 0.92% |
| Bacon, back/middle, unsmoked, grilled (including fat) | 10 | 0.91% |
| Parma Ham | 11 | 0.91% |
| Carbonara pasta sauce | 12 | 0.87% |
| Meatballs in tomato sauce | 9 | 0.87% |
| Venison, roasted or stewed | 7 | 0.85% |
| Scotch pie (Bridie) | 6 | 0.83% |
| Bacon, smoked, fried (fat removed) | 7 | 0.79% |
| McDonalds Cheeseburger | 6 | 0.78% |
| Beef stew with gravy and vegetables | 8 | 0.71% |
| Greggs steak bake | 4 | 0.68% |
| Beef casserole | 10 | 0.66% |
| Luncheon meat, not canned | 10 | 0.65% |
| Scotch broth (soup), canned | 6 | 0.65% |
| Cottage pie (beef), home made | 7 | 0.62% |
| Filled pasta, meat (e.g chicken & chorizo tortelloni) | 4 | 0.56% |
| Pancetta, cooked | 5 | 0.56% |
| Omelette with ham & cheese | 5 | 0.55% |
| Lamb curry, homemade | 5 | 0.54% |
| Steak pie, individual (including steak and kidney) | 8 | 0.54% |
| Cheese and ham sandwich with wholemeal/oatmeal bread | 5 | 0.53% |
| Smoked Sausage | 4 | 0.53% |
| Meat pizza (e.g. Hawaiian, pepperoni, meat feast), takeaway/restaurant | 4 | 0.52% |
| Bacon, back/middle, smoked, grilled (fat removed) | 6 | 0.51% |
| Dairylea Lunchables (includes cheese, meat and crackers) | 1 | 0.49% |
| Spring roll with meat and vegetables | 3 | 0.49% |
| Pepperami or snack salami | 3 | 0.48% |
| Square/Lorne sausage | 1 | 0.48% |
| Roast beef | 10 | 0.47% |
| Bacon, unsmoked, fried (including fat) | 7 | 0.46% |
| Ham and cheese sub roll/baguette | 5 | 0.45% |
| Sausage, fried | 5 | 0.44% |
| Corned beef sandwich with wholemeal/oatmeal bread | 1 | 0.43% |
| Beef sirloin steak, fried | 1 | 0.42% |
| Beef sirloin steak, grilled | 3 | 0.40% |
| Beef bolognese sauce, home made | 8 | 0.38% |
| Baked beans and sausages | 4 | 0.37% |
| Pastrami | 3 | 0.37% |
| Bacon, smoked, fried (including fat) | 5 | 0.36% |
| Corned beef , not canned | 4 | 0.36% |
| Ham salad sandwich with wholemeal/oatmeal bread | 5 | 0.36% |
| Loaded potato skins (e.g with cheese & bacon) | 2 | 0.35% |
| Ready cooked savoury rice (e.g. Uncle Bens) | 5 | 0.35% |
| Lamb chop/cutlet, grilled, fat eaten | 3 | 0.34% |
| Beef and vegetable soup | 3 | 0.33% |
| Ravioli (filled pasta) in tomato sauce, canned | 2 | 0.33% |
| Beef/frying steak | 1 | 0.31% |
| Minced beef, fried | 1 | 0.31% |
| Minced beef, stewed, with vegetables and gravy | 5 | 0.31% |
| Mixed meat kebabs | 2 | 0.31% |
| Pasta carbonara (e.g. spaghetti), ready meal | 3 | 0.31% |
| BLT sandwich with wholemeal/oatmeal bread | 2 | 0.30% |
| Beef sandwich with white/malted bread | 5 | 0.30% |
| Meat risotto (e.g. beef/ham) | 3 | 0.30% |
| Pulled pork | 2 | 0.30% |

Data are from adults (≥16 years) in the 2021 Scottish Health Survey. Food items contributing <0.3% are not shown. Low consumers (>0g to ≤34.5g/d total red and red processed meat), n=824.

Abbreviation: RPM; Red and red processed meat

# **Table 10.** Consumption frequency of RPM items among medium consumers

| **Food Description** | **Unweighted n** | **Weighted %** |
| --- | --- | --- |
| Pork sausage, grilled | 54 | 4.20% |
| Beef burger/hamburger, in a bun, not quarter pounder | 33 | 4.11% |
| Ham sandwich with wholemeal/oatmeal bread | 56 | 3.98% |
| Ham, not smoked | 67 | 3.66% |
| Spaghetti bolognese, home made (pasta and sauce) | 46 | 3.59% |
| Chilli con carne | 42 | 2.81% |
| Meat pizza (e.g. Hawaiian, pepperoni, meat feast) | 30 | 2.73% |
| Ham sandwich with white/malted bread | 39 | 2.72% |
| Bacon, back/middle, unsmoked, grilled (fat removed) | 38 | 2.18% |
| Cheese and ham sandwich with white/malted bread | 27 | 1.92% |
| Beef lasagne | 23 | 1.80% |
| Ham, smoked | 33 | 1.80% |
| Haggis | 17 | 1.56% |
| Steak pie, slice from a large pie (including steak and kidney) | 25 | 1.49% |
| Sausage roll | 22 | 1.42% |
| Ham salad sandwich with wholemeal/oatmeal bread | 10 | 1.36% |
| Pate (e.g. brussels liver pate / duck and orange pate) | 27 | 1.36% |
| Roast beef | 24 | 1.27% |
| Salami | 23 | 1.24% |
| Cheese and ham sandwich with wholemeal/oatmeal bread | 14 | 1.20% |
| Black pudding | 20 | 1.19% |
| Chicken and vegetable soup, homemade | 25 | 1.19% |
| Square/Lorne sausage | 18 | 1.17% |
| Bacon, smoked, fried (including fat) | 12 | 1.03% |
| Bacon, back/middle, smoked, grilled (including fat) | 13 | 0.96% |
| Sausage, fried | 16 | 0.93% |
| Bacon, back/middle, unsmoked, grilled (including fat) | 17 | 0.89% |
| Beef sirloin steak, fried | 7 | 0.88% |
| Beef stew with gravy and vegetables | 12 | 0.85% |
| Chorizo | 13 | 0.83% |
| Beef burger, 100% beef, grilled (no bun) | 7 | 0.79% |
| Bacon sandwich with white/malted bread | 11 | 0.78% |
| Cup a soup, made up | 10 | 0.76% |
| Sausage sandwich with ketchup with white/malted bread or roll | 8 | 0.74% |
| Meat pizza (e.g. Hawaiian, pepperoni, meat feast), takeaway/restaurant | 10 | 0.71% |
| Pasta carbonara (e.g. spaghetti) | 7 | 0.70% |
| Beef lasagne, ready meal | 12 | 0.69% |
| Steak pie, individual (including steak and kidney) | 14 | 0.68% |
| Minced beef, stewed, with vegetables and gravy | 3 | 0.67% |
| Scotch pie (Bridie) | 9 | 0.64% |
| Meat based quiche (e.g. quiche lorraine) | 10 | 0.63% |
| Chicken and bacon sandwich with white/malted bread | 4 | 0.62% |
| Cottage pie (beef), home made | 8 | 0.55% |
| Beef Sausage, grilled | 7 | 0.54% |
| Pork chop, grilled, fat not eaten | 10 | 0.53% |
| Beef stir fry (meat and vegetables) | 8 | 0.52% |
| Luncheon meat/chopped pork, canned (e.g. spam) | 6 | 0.51% |
| Cottage pie (beef), ready meal | 6 | 0.50% |
| Bacon, back/middle, smoked, grilled (fat removed) | 9 | 0.49% |
| Ham salad sub roll/baguette | 8 | 0.48% |
| Lamb curry, homemade | 4 | 0.48% |
| McDonalds Cheeseburger | 3 | 0.48% |
| Roast/braised beef brisket/silverside | 7 | 0.48% |
| Beef sirloin steak, grilled | 7 | 0.46% |
| Breakfast sandwich with wholemeal/oatmeal bread | 2 | 0.46% |
| Beef fillet steak, grilled | 7 | 0.45% |
| Serrano ham | 6 | 0.45% |
| Beef burger, fried (no bun) | 6 | 0.44% |
| Minced beef in gravy | 9 | 0.43% |
| Bacon sandwich with wholemeal/oatmeal bread | 8 | 0.42% |
| Beef burger, grilled (no bun) | 6 | 0.42% |
| Meatballs in tomato sauce | 4 | 0.42% |
| Minced beef, stewed | 8 | 0.41% |
| Scotch egg (including mini/picnic size) | 6 | 0.41% |
| Greggs steak bake | 6 | 0.40% |
| Mixed pizza (e.g. chicken and bacon) | 6 | 0.40% |
| Roast beef slices, pre-packed/deli | 6 | 0.40% |
| Scotch broth (soup), canned | 6 | 0.40% |
| Luncheon meat, not canned | 12 | 0.39% |
| Pork pie | 12 | 0.39% |
| Shepherd's pie (lamb), home made | 8 | 0.39% |
| Beef fillet steak, fried | 7 | 0.38% |
| Sausage casserole | 6 | 0.38% |
| Bacon/gammon joint, boiled (fat removed) | 5 | 0.37% |
| Corned beef , not canned | 7 | 0.37% |
| Minced beef pie (including beef and potato) | 5 | 0.37% |
| Carbonara pasta sauce | 6 | 0.36% |
| BLT sandwich with wholemeal/oatmeal bread | 4 | 0.35% |
| Meatballs fried | 2 | 0.35% |
| Meat samosas | 2 | 0.33% |
| Cheese burger, in a bun, quarter pounder | 7 | 0.32% |
| Meat pizza (e.g. Hawaiian, pepperoni, meat feast), stuffed crust, takeaway/restaurant | 2 | 0.32% |
| Pepperami or snack salami | 4 | 0.32% |
| Veal mince, stewed | 4 | 0.32% |
| Bacon, smoked, fried (fat removed) | 4 | 0.30% |

Data are from adults (≥16 years) in the 2021 Scottish Health Survey.

Food items contributing <0.3% are not shown.

Medium consumers (>34.5g to 70g/d total red and red processed meat consumption), n=784.

Abbreviation: RPM; Red and red processed meat.

# **Table 11.** Consumption frequency of RPM items among high consumers

| **Food Description** | **Unweighted n** | **Weighted %** |
| --- | --- | --- |
| Ham sandwich with white/malted bread | 108 | 5.39% |
| Ham, not smoked | 116 | 3.78% |
| Pork sausage, grilled | 99 | 3.74% |
| Spaghetti bolognese, home made (pasta and sauce) | 80 | 3.43% |
| Ham sandwich with wholemeal/oatmeal bread | 72 | 3.41% |
| Sausage roll | 45 | 2.33% |
| Bacon, back/middle, unsmoked, grilled (fat removed) | 55 | 2.00% |
| Bacon sandwich with white/malted bread | 39 | 1.90% |
| Sausage, fried | 46 | 1.89% |
| Square/Lorne sausage | 52 | 1.86% |
| Beef burger/hamburger, in a bun, not quarter pounder | 39 | 1.84% |
| Meat pizza (e.g. Hawaiian, pepperoni, meat feast) | 33 | 1.80% |
| Chilli con carne | 47 | 1.72% |
| Beef sirloin steak, grilled | 33 | 1.46% |
| Ham, smoked | 41 | 1.43% |
| Black pudding | 30 | 1.35% |
| Pate (e.g. brussels liver pate / duck and orange pate) | 27 | 1.29% |
| Cup a soup, made up | 24 | 1.27% |
| Bacon, smoked, fried (including fat) | 26 | 1.22% |
| Cheese and ham sandwich with white/malted bread | 29 | 1.20% |
| Roast beef | 40 | 1.20% |
| Bacon, back/middle, smoked, grilled (including fat) | 29 | 1.07% |
| Minced beef, stewed | 28 | 1.05% |
| Beef lasagne | 22 | 1.02% |
| Bacon, back/middle, unsmoked, grilled (including fat) | 29 | 0.98% |
| Beef Sausage, grilled | 24 | 0.88% |
| Beef/frying steak | 25 | 0.88% |
| Steak pie, slice from a large pie (including steak and kidney) | 20 | 0.86% |
| Pork pie | 20 | 0.84% |
| Chorizo | 13 | 0.80% |
| Cheese and ham sandwich with wholemeal bread | 19 | 0.78% |
| Pork fillet (tenderloin) | 23 | 0.78% |
| Luncheon meat/chopped pork, canned (e.g. spam) | 10 | 0.76% |
| Steak pie, individual (including steak and kidney) | 15 | 0.75% |
| Pork chop, fried | 19 | 0.74% |
| Pork chop, grilled, fat not eaten | 25 | 0.74% |
| Beef sirloin steak, fried | 22 | 0.71% |
| McDonald's Big Mac | 8 | 0.71% |
| Meat pizza (e.g. Hawaiian, pepperoni, meat feast), takeaway/restaurant | 14 | 0.69% |
| Minced beef, fried | 22 | 0.69% |
| Chicken and vegetable soup, homemade | 18 | 0.65% |
| Beef stew with gravy and vegetables | 23 | 0.64% |
| Beef burger, grilled (no bun) | 15 | 0.57% |
| Lamb curry, homemade | 10 | 0.56% |
| Veal mince, stewed | 10 | 0.56% |
| Bacon sandwich with wholemeal/oatmeal bread | 18 | 0.53% |
| Sausage casserole | 9 | 0.53% |
| Beef fillet steak, grilled | 13 | 0.52% |
| Glazed baked gammon | 12 | 0.50% |
| Pork chop, grilled, fat eaten | 9 | 0.50% |
| Lamb chop/cutlet, grilled, fat eaten | 11 | 0.49% |
| Bacon, unsmoked, fried (including fat) | 17 | 0.47% |
| Ham salad sandwich with white/malted bread | 11 | 0.47% |
| Pasta carbonara (e.g. spaghetti) | 8 | 0.47% |
| Haggis | 16 | 0.45% |
| Ham, low fat | 17 | 0.45% |
| McDonalds Cheeseburger | 6 | 0.45% |
| Salami | 15 | 0.45% |
| Sausage sandwich with ketchup with white/malted bread or roll | 11 | 0.45% |
| Luncheon meat, not canned | 12 | 0.44% |
| Beef and vegetable soup | 11 | 0.41% |
| Chicken and bacon sub roll/baguette | 8 | 0.41% |
| Beef curry, takeaway | 5 | 0.40% |
| Bacon/gammon joint, boiled (fat removed) | 8 | 0.38% |
| Burger King bacon cheeseburger | 3 | 0.38% |
| Chicken mayonnaise sandwich fillers (e.g. chicken and sweetcorn, tikka, coronation) | 9 | 0.38% |
| Bacon, back/middle, smoked, grilled (fat removed) | 11 | 0.37% |
| Beef sandwich with white/malted bread | 11 | 0.37% |
| Corned beef sandwich with white/malted bread | 15 | 0.37% |
| Chicken and bacon wrap | 7 | 0.36% |
| Pigs in blankets | 3 | 0.36% |
| Scotch broth (soup), canned | 9 | 0.36% |
| Scotch pie (Bridie) | 8 | 0.36% |
| Minced beef pie (including beef and potato) | 8 | 0.35% |
| Beef bolognese sauce, home made | 9 | 0.34% |
| Beef stir fry (meat and vegetables) | 8 | 0.34% |
| Corned beef , not canned | 14 | 0.34% |
| Meatballs in tomato sauce | 10 | 0.34% |
| Smoked Sausage | 8 | 0.34% |
| Ham salad sandwich with wholemeal/oatmeal bread | 12 | 0.33% |
| Hot dog/frankfurter with sauce in a bun | 5 | 0.33% |
| Minced beef, stewed, with onion | 11 | 0.32% |
| Beef fillet steak, fried | 15 | 0.31% |
| Cottage pie (beef), home made | 7 | 0.31% |
| Beef chow mein, stir fry (with noodles) | 7 | 0.30% |
| Cheese burger, in a bun, quarter pounder | 7 | 0.30% |
| Pork/pork and beef meatballs, grilled | 8 | 0.30% |

Data are from adults (≥16 years) in the 2021 Scottish Health Survey.

Food items contributing <0.3% are not shown.

High consumers (>70g/d total red and red processed meat), n=886

Abbreviation: RPM; Red and red processed meat
